# Prognostic Significance of Transverse Relaxation Rate (R2*) in Blood Oxygenation Level-Dependent Magnetic Resonance Imaging in Patients with Invasive Breast Cancer

**DOI:** 10.1371/journal.pone.0158500

**Published:** 2016-07-06

**Authors:** Hye Young Choi, Eun Sook Ko, Boo-Kyung Han, Eun Ju Kim, Sun Mi Kim, Yaeji Lim, Rock Bum Kim

**Affiliations:** 1 Department of Radiology, Gyeongsang National University Hospital and Graduate School of Medicine, Jinju, Korea; 2 Department of Radiology, Samsung Medical Center, Sungkyunkwan University School of Medicine, Seoul, Korea; 3 Department of Clinical Science, MR, Philips Healthcare Korea, Seoul, Korea; 4 Department of Radiology, Seoul National University Bundang Hospital, Seongnam, Korea; 5 Biostatistics and Clinical Epidemiology Center, Samsung Medical Center, Seoul, Korea; 6 Regional Cardiocerebrovascular Center, Gyeongsang National University Hospital, Jinju, Korea; The University of Hong Kong, CHINA

## Abstract

**Objective:**

To examine the relationship between magnetic resonance transverse relaxation rate (R2*) and prognostic factors.

**Materials and Methods:**

A total of 159 women with invasive ductal carcinomas (IDCs) underwent breast magnetic resonance imaging (MRI) including blood oxygenation level-dependent (BOLD) sequence at 3 T. The distribution of the measured R2* values were analyzed, and the correlation between R2* and various prognostic factors (age, tumor size, histologic grade, lymphovascular invasion, and axillary lymph node status, as well as expression of estrogen receptor, progesterone receptor, human epidermal growth factor receptor 2, p53, and Ki-67) were retrospectively assessed using patient medical records.

**Results:**

The baseline R2* values of the IDCs were very heterogeneous with wide range among the patients. The mean R2* value was (32.8 ± 14.0) Hz with a median of 29.3 Hz (range 13.5–109.4 Hz). In multivariate analysis, older age was associated with decreased R2* value (*P* = 0.011) and IDCs with p53-overexpression showed higher R2* values than those without p53-overexpression group (*P* = 0.031). Other prognostic factors were not significantly correlated with R2* value.

**Conclusion:**

In this study, R2* values were significantly correlated with age and expression of p53. Further studies are necessary to determine the prognostic value of BOLD-MRI.

## Introduction

It is well established that hypoxia increases the malignant potential of tumors and promotes their viability by altering gene expression patterns via multiple mechanisms [1, 2]. Because hypoxia is an important biological characteristic of tumors, which leads to treatment resistance and enhanced tumor progression, imaging techniques that reflect tumor hypoxia may provide noninvasive prognostic prediction and guide cancer therapies [2, 3].

A number of hypoxia imaging techniques have been studied. Although historically used as the reference standard for direct measurement of human tumor hypoxia, the Eppendorf histograph system is invasive and cannot be applied to all tumor types [4]. In addition, immunohistochemistry of endogenous hypoxic markers (e.g., hypoxia-inducible factor 1 [HIF-1]) or exogenous nitroimidazole requires biopsy or surgery [5]. Positron emission tomographic (PET) imaging and magnetic resonance imaging (MRI) are noninvasive, widely available techniques used to assess tumor hypoxia *in vivo* [2, 3, 6, 7]. Although many studies have suggested PET as a promising non-invasive imaging modality for visualizing tumor oxygenation *in vivo*, this modality has some limitations, including low spatial resolution, inaccuracy of anatomy, administration of an exogenous radioactive tracer, etc. [2, 6]. However, MRI is widely used for diagnosis, breast tumor staging, prognosis prediction, and monitoring of treatment response, without the limitations associated with PET. Therefore, development of a noninvasive MRI-based, oxygen-sensitive test seems attractive. In the recent decade, functional MRI, including dynamic contrast-enhanced (DCE) MRI and blood oxygenation level-dependent (BOLD)-MRI (also called intrinsic susceptibility-weighted MRI) has shown potential as a reliable imaging modality for measuring tumor hypoxia [2, 3, 7–9]. BOLD-MRI exploits the paramagnetic properties of deoxyhemoglobin in erythrocytes to create contrast in MR images; unlike DCE-MRI, it does not require administration of exogenous contrast material, and produces images with high temporal and spatial resolution and reproducibility [10]. Several preclinical studies including a rat model of chemically induced mammary tumors have shown an association between BOLD-MRI signal response and tumor oxygenation [11, 12].

To date, there have been few reports on BOLD-MRI of human breasts, including a very small number of breast tumors [13–16]. Recently, a study of BOLD-MRI in 104 breast invasive ductal carcinomas (IDCs) showed that resonance transverse relaxation rate (R2*) values were correlated with levels of HIF-1α, an endogenous hypoxic marker, and two prognostic factors, axillary lymph node metastasis and Ki-67 expression [17]. However, to determine the prognostic value of BOLD-MRI in human invasive breast cancer, further studies with larger sample sizes and, thus, higher statistical power are required. Therefore, our study examined the relationship between R2* values and other well-established clinicopathological prognostic factors in a larger study population.

## Materials and Methods

### Ethics statement

This research was approved by the Institutional Review Board of Samsung Medical Center (SMC 2016-03-039). Informed consent was waived because the data collection and analysis in this study were performed retrospectively and anonymously.

### Patients

From December 2013 to May 2014, a total of 344 consecutive patients with pathologically confirmed breast cancer underwent breast MRI including BOLD sequence at 3 T. To evaluate the relationship between R2* values and prognostic factors under homogeneous conditions, the lesions included in our study were restricted to those that met the following criteria: (1) a final pathological diagnosis of invasive ductal carcinoma not otherwise specified (IDC NOS); (2) presented as a mass on MRI; and (3) tumor size of at least 1.0 cm in maximum diameter. We excluded cases for which MRI was performed after diagnosis by means of vacuum-assisted or excisional biopsy (*n* = 23). Patients received neoadjuvant chemotherapy (n = 40), and those who had a mass not visible in BOLD-MRI due to not being covered (n = 6) were also excluded. Therefore, a total of 159 female cancer patients aged 32–84 years (mean, 50.7 years) comprised the study group.

The established clinicopathological variables of breast cancer clinical outcome and response to therapy include age, tumor size, histologic grade, lymphovascular invasion, axillary lymph node status, and expression of ER, PR, HER2, p53, and Ki-67 [18]. These clinicopathological characteristics were recorded by reviewing patient medical records.

### Breast MRI Protocol

Breast MRI was performed using 3 T Achieva scanners (Philips Medical Systems, Best, The Netherlands) with a dedicated bilateral phased array breast coil. Images of both breasts were acquired with the patient in the prone position. Breast BOLD-MRI was included in the routine MRI protocol and baseline R2* values were determined in patients not receiving oxygen. The routine breast MRI examination included turbo spin-echo T1- and T2-weighted sequences and a three-dimensional dynamic contrast-enhanced sequence. Before injection of the contrast agent, BOLD-MRI was performed using a multiple fast-field echo sequence and two protocols with different gradient echoes (15 and 20) to obtain T2*-weighted images in the axial planes. Because blood flow also affects contrast in MR images, we performed BOLD-MRI using a multi-gradient-echo sequence to eliminate its effects. In the protocol using a gradient echo of 15, the repetition time (TR) was 464 ms, the initial echo time (TE) was 1.48 ms, and delta TE (the time between echoes) was 1.4 ms. In the protocol using a gradient echo of 20, the TR was 577 ms, the initial TE was 1.07 ms, and the delta TE was 1.3 ms. In both protocols, scanning began at 10 ms with a 1.4-ms increment. The remaining parameters for both protocols were as follows: flip angle, 15°; reconstructed matrix, 224 × 224; sensitivity-encoding factor, 2; number of signals acquired, 1; field of view, 320 × 250 mm; slice thickness, 3 mm; interslice gap, 1 mm; and slice number, 20.

### Image Analysis

**Fig 1:**
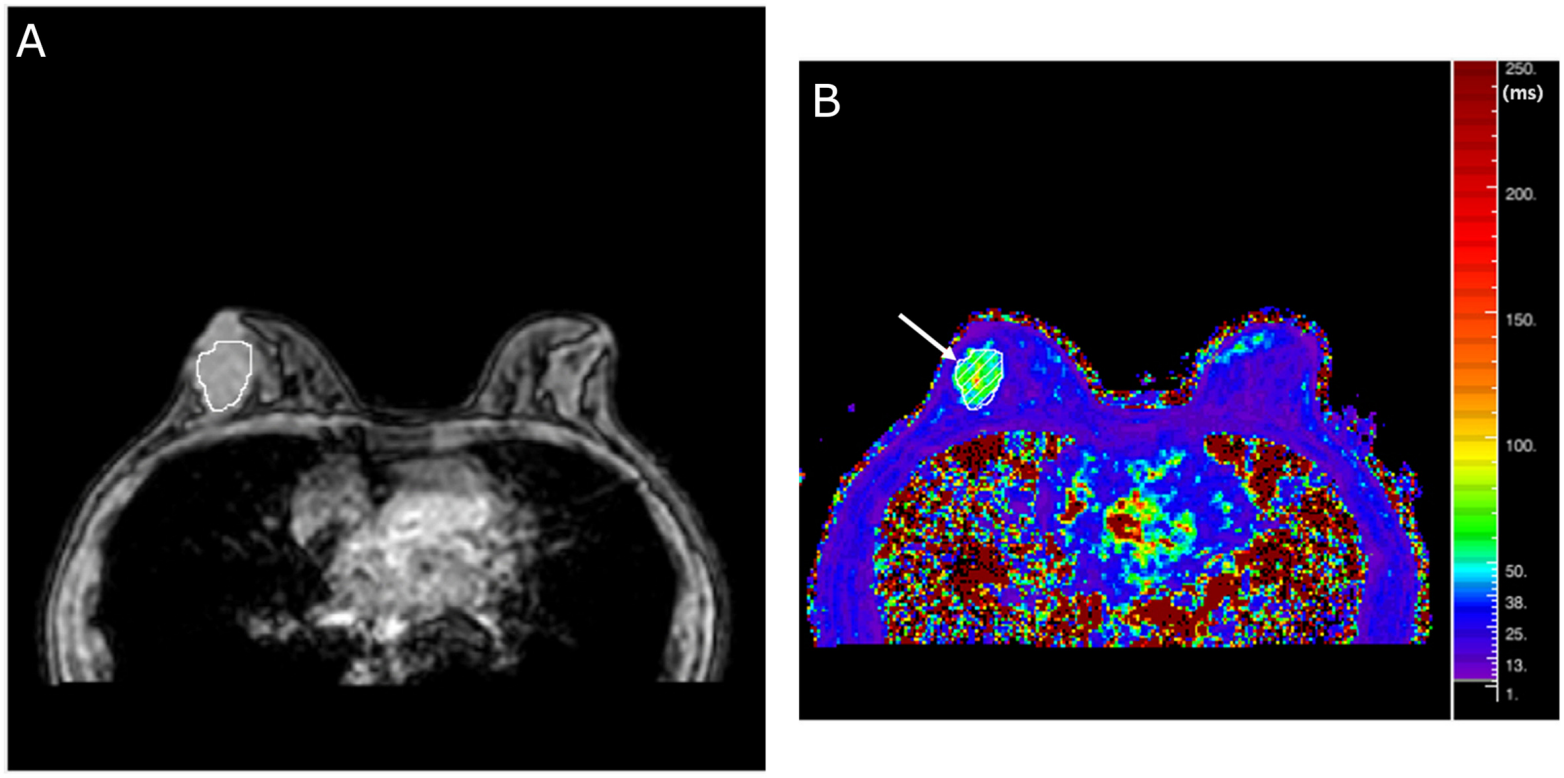
(**A**) Axial T2*-weighted image and (**B**) color-coded R2* map from BOLD-MRI of a 34-mm-diameter grade 1 IDC (T2N1M0) in a 48-year-old woman. The R2* map shows heterogeneous signal intensity in the tumor (arrow). The red color represents the highest R2* values, indicating the highest concentration of deoxyhemoglobin; the blue color represents the lowest R2* values, indicating the lowest concentration of deoxyhemoglobin. The mean R2* value was 56.08 Hz.

After data acquisition, all BOLD-MRI scans were transferred to a workstation for analysis using manufacturer-supplied software (PRIDE Relaxation Maps Tool, version 2.1.1, Philips Healthcare). This software program generates a set of color-coded axial parametric images of R2* values (1/s) from a voxel basis to an exponential function describing the expected signal decay as a function of TE and solving for the unknown value of R2*. On the R2* map, red areas represent the highest R2* values, reflecting the highest concentration of deoxyhemoglobin, whereas blue areas represent the lowest R2* values and lowest deoxyhemoglobin concentrations (Fig 1). Data analysis was performed by and required the consensus of two dedicated breast radiologists with 6 and 10 years of experience in breast MRI, respectively. The radiologists were blinded to the clinicopathological information.

Quantitative analysis was performed using routine breast MRI scans as the anatomic references and placing regions of interest (ROI) on the color-coded parametric R2* maps. R2* values in tumors were automatically calculated. In cases with multifocal or multicentric breast cancers, the R2* value of the largest tumor was recorded. The ROIs were manually drawn in the solid portion of the tumor to encompass as much of the lesion as possible on the slice in which the tumor showed the greatest diameter, while avoiding cystic or necrotic regions and normal breast parenchyma. An average of two or three measurements per lesion was performed. The ROIs included at least 25 pixels (mean, 268.6 pixels).

### Statistical Analysis

Before testing the relationship between R2* values and prognostic factors, the Shapiro-Wilk test was performed to assess the normality of the R2* values. Because R2* values of breast cancer were not normally distributed, logarithmic transformation of the skewed data was used. As the log transformed R2* value did not satisfy the normality assumption, we subtracted 10 from the R2* value before log transformation. The natural log of (R2* value −10) with normal distribution was applied to the following statistical analyses. Pearson’s correlation analysis was used to evaluate the association of log transformed R2* value with patient age and tumor size. Student’s t-test and one-way ANOVA (analysis of variance) were used to evaluate differences in log transformed R2* value according to prognostic factors. For multivariate analysis, multiple linear regression analysis with stepwise method (using *P* value < 0.1 as entry criterion, *P* value ≥ 0.1 as removal criterion) was performed to identify variables independently associated with log transformed R2* value. Multicollinearity was checked using variance inflation factor (VIF). There were no variables with VIF > 10.

All analyses were performed using statistical software (SPSS for Windows, version 21.0; IBM Corp., Armonk, NY, USA) with *P* < 0.05 (two-sided test) defined as the threshold for a statistically significant difference.

## Results

### Correlations Between R2* Values and Clinicopathological Prognostic Factors

**Fig 2:**
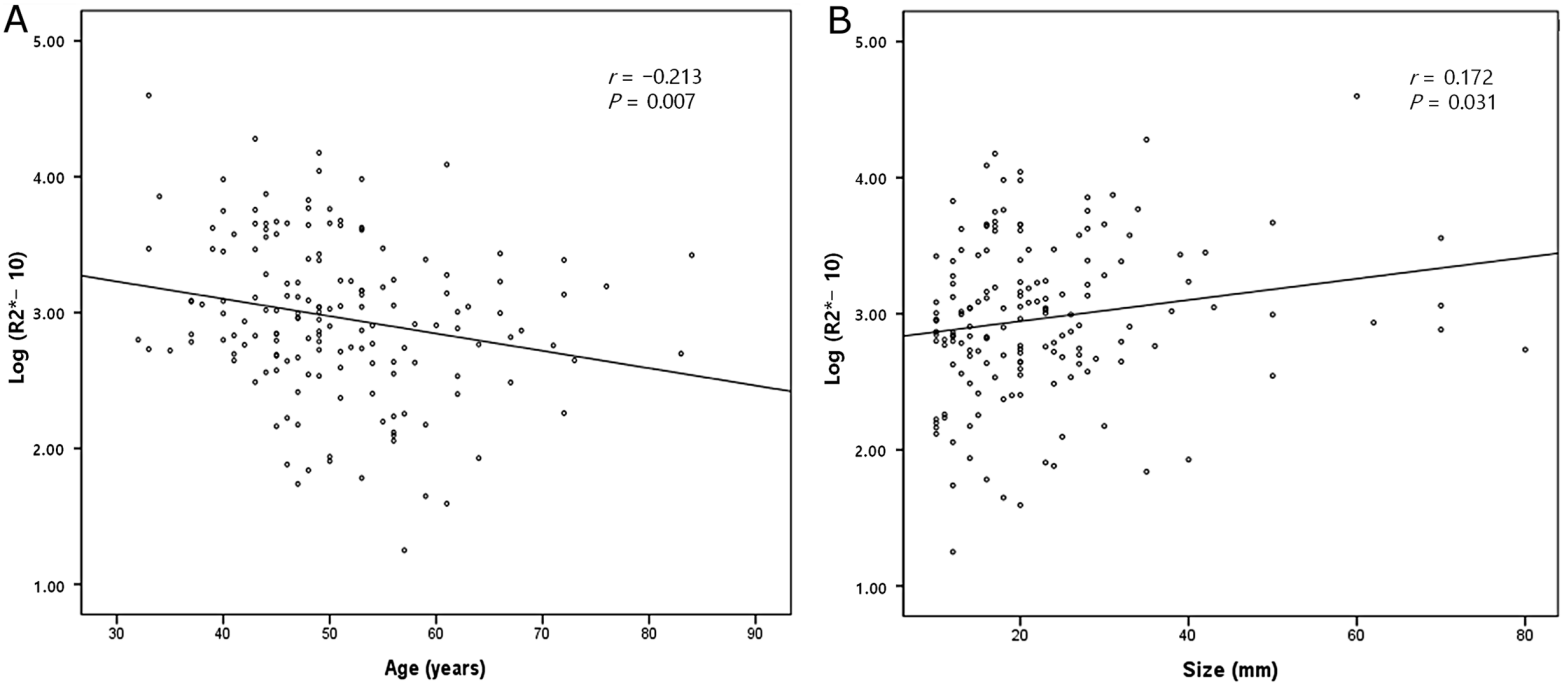
Scatter plots showing Pearson’s correlation of log transformed R2* value with (A) age and (B) tumor size. Log transformed R2* value correlates weakly with age and tumor size (correlation coefficient, *r* = -0.213 and 0.172, respectively).

The study population consisted of 159 female patients (mean age 50.7 years, SD ± 9.6; range 32.0–84.0 years) with 159 IDCs. The mean diameter of the lesions determined via MRI was 22.5 mm (SD ± 12.6; range 10–80 mm). Axillary lymph node metastases were detected in 71 (44.7%) patients. The histopathologic features of the tumors are shown in Table 1. The R2* maps of the tumors showed a relatively heterogeneous signal (Fig 1). The mean R2* value was 32.8 Hz (SD ± 14.0) with a median of 29.3 Hz (range 13.5–109.4 Hz). Pearson’s correlation analysis showed that log transformed R2* value correlated weakly with age (*r* = −0.213, *P* = 0.007) and tumor size (*r* = 0.172, *P* = 0.031) (Fig 2). R2* values decreased slightly as age increased and increased slightly as tumor size increased. The R2* values in three histologic grade groups were compared. Although grade 1 tumors had the lowest R2* values, the differences between the three groups were not significant (*P* = 0.095). Univariate analysis showed that R2* values were significantly correlated with some prognostic factors (age, tumor size and expression of p53), but not others (Fig 2 and Table 1).

**Table 1:** Correlations between R2* Values and Clinicopathological Prognostic Factors.

	N (%)	R2* (Hz)	R2* (Hz)	*P* value ^†^
		Mean ± SD	Median (Range)	
Age at diagnosis (years)				0.042^‡^
30–39	13 (8.2)	40.3 ± 23.0	31.8 (25.2–109.4)	
40–49	72 (45.3)	34.6 ± 13.9	30.0 (15.7–82.1)	
50–59	48 (30.2)	29.4 ± 11.4	27.6 (13.5–63.6)	
60–69	18 (11.3)	30.0 ± 11.9	28.3 (14.9–69.7)	
≥70	8 (5.0)	30.2 ± 7.7	29.4 (19.6–40.7)	
Tumor size (mm)				0.212^‡^
≤20	94 (59.1)	31.8 ± 13.1	27.9 (13.5–75.2)	
20–50	59 (37.1)	33.2 ± 12.2	30.9 (16.3–82.1)	
>50	6 (3.8)	44.6 ± 32.5	28.8 (25.4–109.4)	
Histologic grade				0.095
1	41 (26.1)	31.0 ± 11.2	28.8 (14.9–56.0)	
2	63 (40.1)	31.5 ± 15.5	28.0 (13.5–109.4)	
3	53 (33.8)	35.6 ± 13.8	32.4 (16.6–75.2)	
Lymphovascular invasion				0.946
No	82 (51.6)	33.1 ± 15.0	29.3 (15.2–109.4)	
Yes	77 (48.4)	32.5 ± 12.9	29.2 (13.5–82.1)	
Axillary lymph node metastasis				0.748
No	86 (54.8)	32.6 ± 14.6	28.4 (14.9–109.4)	
Yes	71 (45.2)	33.0 ± 13.3	30.0 (13.5–82.1)	
Estrogen receptor				0.062
Negative	23 (14.5)	38.1 ± 15.5	35.3 (15.2–75.2)	
Positive	136 (85.5)	31.9 ± 13.6	28.3 (13.5–109.4)	
Progesterone receptor				0.550
Negative	34 (21.4)	34.2 ± 14.1	31.2 (15.2–75.2)	
Positive	125 (78.6)	32.4 ± 14.0	28.3 (13.5–109.4)	
HER2 amplification				0.742
Negative	125 (79.1)	33.0 ± 14.5	29.4 (13.5–109.4)	
Positive	33 (20.9)	31.9 ± 12.3	26.9 (15.2–69.7)	
p53				0.016
Negative	117 (75.5)	31.6 ± 13.8	28.3 (13.5–109.4)	
Positive	38 (24.5)	37.2 ± 14.3	32.7 (16.7–75.2)	
Ki-67				0.055
<14%	75 (47.2)	31.1 ± 14.5	28.4 (13.5–109.4)	
≥14%	84 (52.8)	34.3 ± 13.4	30.9 (15.9–75.2)	

HER2 = human epidermal growth factor receptor 2, SD = standard deviation

^†^ These are results by Student’s t-tests and one-way ANOVA to evaluate differences in log (R2* value −10) according to prognostic factors.

^‡^ These analyses of categorical data have yielded results different from those of Pearson’s correlation analysis with continuous data (shown in Fig 2).

### Multivariate Analysis

Multiple linear regression analysis with a stepwise method was performed to identify variables independently associated with log transformed R2* value. Age and expression of p53 were independently associated with log transformed R2* value (*P* = 0.011and 0.031, respectively) (Table 2). As age increased, R2* values decreased slightly. IDCs with p53-overexpression showed slightly higher R2* values than those without p53-overexpression. Other prognostic factors failed to reach statistical significance.

**Table 2:** Multiple linear regression analysis of variables independently associated with R2*.

Variable	β	SE	95% CI	*P* value
Age	-0.01	0.005	-0.02, 0.00	0.011
Tumor size	0.01	0.00	0.00, 0.01	0.078
Histologic grade				
1				
2	-0.07	0.12	-0.30, 0.17	0.582
3	0.04	0.16	-0.27, 0.35	0.829
Estrogen receptor				
Negative				
Positive	-0.14	0.15	-0.43, 0.15	0.341
p53				
Negative				
Positive	0.23	0.11	0.02, 0.44	0.031
Ki-67				
<14%				
≥14%	0.09	0.12	-0.14, 0.32	0.436

β = regression coefficient, SE = standard error, CI = confidence interval

## Discussion

BOLD-MRI is a potential candidate for an optimal imaging modality to assess tumor hypoxia. However, the pathophysiologic–imaging correlates and the prognostic value of BOLD-MRI have not been well established in human invasive breast cancers. A recent study of BOLD-MRI in 104 breast IDCs showed that R2* values correlated with the levels of HIF-1α, an endogenous hypoxic marker (ρ = 0.516, *P* = 0.000), indicating that faster R2* values were associated with increased hypoxia [17]. However, R2* values were not significantly correlated with most prognostic factors except for axillary lymph node metastasis and Ki-67 expression. Based on prior studies, we assessed the correlation between R2* values and other clinicopathological prognostic factors in human invasive breast cancer in a relatively larger population (n = 159). In our study, most prognostic factors except for age and expression of p53 were not significantly correlated with R2* value.

In our study, the baseline R2* values of the IDCs and normal breast tissue were very heterogeneous within the every single tumor and among the patients. This finding is similar to reports from previous studies [12, 17]. Because breast cancer is a heterogeneous group of tumors with variable clinical course, treatment response, and prognosis, the tumors presumably have varying degrees of hypoxia [19]. However, wide heterogeneity in the baseline R2* values of the tumors may be affected by the oxygenation status of background parenchyma in patients. We suspect our result that there was no significant correlation between R2* and other prognostic factors partially was caused from those variability of baseline R2* of normal tissue and cancerous tissue. Therefore, as with ADC normalization in diffusion-weighted imaging [20, 21], R2* normalization using normal glandular tissue might be needed to eliminate the effects of variations in background parenchyma.

Our study showed that age was a variable independently associated with R2* (*P* = 0.011). R2* values decrease slightly with increasing age. If baseline R2* reflects the tissue oxygenation status and tumors with slower R2* are less hypoxic, then it is likely that as age increases, tumors are slightly less hypoxic. Several studies have reported that older women have less aggressive breast cancer, including a higher frequency of lower grade tumors and positivity for hormone receptors [22], although our results showed that tumor grade and hormone receptor status were not significantly correlated with R2 * values. In our study, although tumors of lower histologic grade had lower mean R2* values, the differences between the three tumor grade groups were not significant. A previous study [23] reported a significant inverse correlation between R2* values and tumor grade of human breast IDCs; however, another more recent study of human breast cancer [17] and a study of rat mammary tumors [12] did not. Axillary lymph node metastasis is one of the most important factors in predicting prognosis in breast cancer patients [24]. A recent study [17] reported that patients with positive axillary lymph nodes had significantly higher R2* values than those without metastases. Although our study also observed slightly higher mean R2* values of breast tumors in patients with axillary lymph node metastases than those without metastases, the difference between patients with negative and positive axillary lymph node metastases was not significant. R2* values are affected by tissue perfusion as well as deoxyhemoglobin concentration. Although expression of ER, PR, and HER2 are associated with angiogenesis and perfusion [25, 26], our study found no significant correlation between R2* values and ER, PR, or HER2 expression. These results are consistent with the findings of a recent study [17]. Unlike the recent study [17], our study revealed there was a significant correlation between R2* and p53 expression. IDCs with p53-overexpression showed slightly higher R2* values than those without p53-overexpression. One of the well-known tumor suppressor genes, p53, mediates cell cycle progression, cell senescence, cell differentiation, DNA repair and apoptosis [27]. Although the current evidence is insufficient for p53 to be an independent prognostic marker, recent studies suggest that p53 overexpression might serve as a specific prognostic marker in a specific subgroup of patients with breast cancer [28, 29]. The Ki-67 labeling index, a measure of tumor cell proliferation rate, is a key prognostic factor [30]. While previous studies [17, 31] reported Ki-67 labeling correlated with hypoxia in breast carcinoma, our study found no significant correlation between these parameters.

Our study had several limitations. First, we did not compare R2* values with direct measurements of tumor hypoxia or partial oxygen pressure (pO_2_). BOLD-MR imaging is based on the assumption that oxygenation of hemoglobin is directly proportional to arterial pO_2_. However, it is not a direct measure of tissue pO_2_, but instead reflects deoxyhemoglobin content and may be confounded by tissue blood perfusion, static tissue components, and other factors such as intrinsic T1 and T2 values, and external field inhomogeneities [10]. Negative correlations between baseline R2* and blood volume have been shown in human breast tumors [32, 33]. Padhani et al. suggested that the ability of baseline R2* to reflect tumor hypoxia requires simultaneous assessment of blood volume/perfusion in the tumors, representing functional status of the vasculature [32]. Second, we did not assess the intra- or interobserver variability in the calculation of R2* values. ROIs were manually determined, which may have resulted in errors owing to improper or variable ROI placement on the R2* maps. In our study, the same observer delineated ROIs in the consensus of two radiologists in order to reduce interobserver variability. However, an earlier study showed that the R2* measurements obtained via BOLD-MRI are highly reliable [17]. Therefore, the results of our study may not be greatly affected by our R2* measurement method. Third, our study only included IDCs. Therefore, the relationship between R2* and tumor hypoxia in other histological types of breast cancer was not addressed and requires further investigation. Fourth, we measured the R2* only in the single representative slice not whole-tumor.

In conclusion, our results indicate that R2* is significantly correlated with age and expression of p53. Further studies are necessary to determine the prognostic value of BOLD-MRI. R2* normalization using normal glandular tissue and simultaneous assessment of blood volume/perfusion may promote the clinical application of BOLD-MRI by offering more accurate reflection for tissue hypoxia, thereby leading to improvements in treatment planning and response monitoring in patients with breast cancer.
